# Assessing vaccine hesitancy among nurses in Quebec, Canada using the professionals’ vaccine confidence and behaviors questionnaire: a cross sectional survey

**DOI:** 10.1016/j.ijnsa.2026.100722

**Published:** 2026-10-27

**Authors:** Eve Dubé, Marie-Eve Trottier, Lisa Fressard, Philip White, Arnaud Gagneur, Pierre Verger

**Affiliations:** a Department of Anthropology, Laval University, Pavillon Charles-De Koninck, bureau 3431, 1030, avenue des Sciences-Humaines, Université Laval, Québec G1V 0A6, Canada; b Department of Immunization, CHU de Québec Laval University, 2705, boulevard Laurier, Québec, QC G1V 4G2, Canada; c ORS Paca, Southeastern Health Regional Observatory, Faculté des sciences médicales et paramédicales, 27 Bd Jean Moulin, 13005, Marseille, France; d Population Health Sciences Institute, University of Newcastle, Baddiley-Clark Bldg, Newcastle upon Tyne NE2 4AX, United Kingdom; e Département de Pédiatrie, Unité de Néonatalogie, CHUS Fleurimont, 3001, 12ième avenue nord, SHERBROOKE (QC) J1H 5N4, Canada; f Unité des Virus Émergents (UVE: Aix-Marseille Univ, Università di Corsica, IRD 190, Inserm 1207, IRBA), Marseille, France

**Keywords:** Health care providers, Vaccine hesitancy, Vaccine promotion, Work conditions, Vaccine confidence, Institutional trust

## Abstract

**Background:**

Vaccine hesitancy remains a major global health challenge, further intensified by the COVID-19 pandemic through widespread misinformation and declining trust in institutions. Healthcare providers, particularly nurses in Quebec (Canada), play a pivotal role in promoting vaccine uptake, yet hesitancy within this group may undermine public confidence and vaccination coverage.

**Objective:**

This study aimed to assess nurses’ vaccination attitudes and behaviours in Québec and examine the influence of working conditions on these outcomes**.**

**Design, setting and participants:**

A cross-sectional online survey was conducted among 1428 nurses using the short French version of the Professionals’ Vaccine Confidence and Behaviors Questionnaire (Pro-VC-Be) questionnaire, a validated tool that measures vaccine confidence, trust in authorities, proactive efficacy (i.e., healthcare professionals’ sense of competence and active commitment to promoting vaccination among their patients) and vaccination-related behaviours (i.e., recommendations of specific vaccines to patients and self-vaccination). Additional items assessed perceived working conditions and perceived work degradation since the COVID-19 pandemic.

**Method:**

Descriptive analyses, regression models and structural equation modeling (SEM) were performed.

**Results:**

A total of 1320 questionnaires were analyzed. Nurses showed high levels of vaccine confidence (mean = 81.5/100) and trust in authorities (80.7/100), but lower proactive efficacy (48.9/100) and vaccination-related behaviours, including vaccine recommendations (40.9/100 and 41.1/100). Simple linear regression models show positive “immunization resources” (i.e., vaccine confidence, trust in authorities, proactive efficacy together in a same scale) were strongly associated with increased vaccine-related behaviours and self-vaccination (p < 0.0001). Structural equations model shows that proactive efficacy emerged as a significant direct predictor of vaccine recommendation (path coefficient = 0.7) while trust in authorities was a significant predictor of vaccine confidence (path coefficient = 0.99). Structural equation modeling (SEM) analyses further highlighted significant indirect pathways linking working conditions to vaccine recommendation behaviours. Multiple linear regression models show that trust in authorities and proactive efficacy were positively associated with perceived working conditions. Trust and efficacy were negatively associated with perceived deterioration of work conditions during the pandemic while reluctant confidence was positively associated with perceived deterioration of work conditions.

**Conclusion:**

Despite generally favorable attitudes toward vaccination, gaps persist in vaccination practices among nurses. Findings suggest that beyond individual beliefs, structural and organizational factors, particularly working conditions, play a critical role in shaping vaccine behaviours. Improving workplace environments and reinforcing institutional trust may represent key levers to strengthen vaccine advocacy among nurses and ultimately enhance population-level vaccination uptake.

**What is already known**

• Healthcare providers’ recommendations are a major determinant of vaccine uptake in the general population.
• Healthcare providers, including nurses, may exhibit vaccine hesitancy and be less likely to recommend vaccination especially if they perceive having difficult working conditions

**What this paper adds**

• The Professionals’ Vaccine Confidence and Behaviour questionnaire enabled the assessment of multiple determinants of behaviours related to vaccine confidence among nurses.
• Drivers of nurses’ vaccination behaviours were vaccine confidence, trust in authorities, and proactive efficacy.

## 1 Background

Lack of vaccine confidence and misinformation about vaccines were identified as the most significant barriers in achieving the objectives of the World Health Organization (WHO) Immunization Agenda 2030(“Weekly Epidemiological Record, 2025, vol. 100, 23 [full issue],” n.d.). The COVID-19 pandemic has generated important challenges through the combined effects of an overabundance of information about vaccines, including mis- and disinformation, and increasing social and political polarization, making it difficult for patients to know what health information is reliable and trustworthy(“Infodemic,” n.d.).

In this context, healthcare providers play a critical role in promoting vaccine uptake through strong and personalized recommendations from trusted providers(Lin et al., 2021a). In Canada, nurses play a pivotal role in immunization, as they are mainly responsible for administering vaccines to patients. Thus, maintaining high levels of vaccine confidence among nurses is essential to sustaining high vaccination coverage in the population, but also to ensuring their own vaccination. Low vaccination coverage among nurses heightens their risk of infection, potentially affecting their health, workforce availability, and patient safety, whilst placing additional strain on already overburdened Canadian healthcare systems(Heyerdahl et al., 2022; Nguyen and Bochnak, 2024, p. 124).

Over the past two decades, numerous studies have documented vaccine hesitancy among healthcare providers across different contexts and vaccine types(Lin et al., 2021a; Paterson et al., 2016a; Peterson et al., 2022a; Prieto-Campo et al., 2024; Verger et al., 2022a, 2022b). Research examining determinants of vaccine hesitancy among healthcare providers consistently highlights the importance of several factors as key drivers of vaccine acceptance, including vaccine-related knowledge, social and professional support, and trust in health authorities and the pharmaceutical industry(Larson et al., 2018; Paterson et al., 2016a; Verger et al., 2022a). Although the majority of nurses view vaccination positively, different studies have shown that a subset remains hesitant and may avoid recommending vaccines or provide ambiguous, misleading, or negative information to patients(Cadeddu et al., 2021; Dubé et al., 2018; Larson et al., 2014; Le Breton et al., 2025; Schmid et al., 2017).

Furthermore, the COVID-19 pandemic profoundly altered healthcare organizations and working conditions, with those involved in vaccination particularly affected by rapid and ongoing changes in policies, guidelines, and clinical practice. Vaccinating nurses were frequently at the forefront of implementing new vaccination strategies and public health directives and, as a result, may have faced additional uncertainties and challenges that could contribute to the erosion of trust(Courbage and Peter, 2021; Kwon and Choi, 2025; Shuster and Lubben, 2022). As Flynn has described, vaccine providers must continuously “marry clinical judgment with national standards,” highlighting the central importance of trust in health authorities and governing institutions (Brownlie and Howson, 2006; Flynn, 2002; Verger and Dubé, 2020). However, during the pandemic, trust in governments and public health authorities declined among both the general population and healthcare providers in Canada and internationally(Garrison et al., 2023; Herati et al., 2023; Karlsson et al., 2023; Lowe et al., 2022; Zhu et al., 2024). Notably, early in the pandemic, Canadian nurses reported lower confidence in COVID-19 vaccines compared with other healthcare providers(Dzieciolowska et al., 2021). Emerging evidence also suggests that the pandemic placed significant moral and emotional burdens on healthcare providers, including experiences of moral injury and psychological distress(Brosset et al., 2025; Nicholas et al., 2020; Spilg et al., 2022; Zahiriharsini et al., 2022). Many faced constrained working conditions, limited resources, and insufficient time to deliver care aligned with their professional standards. Others encountered increasing numbers of patients who were reluctant, distrustful, or openly resistant to vaccination. In a context marked by heightened misinformation and disinformation about COVID-19 and vaccines, these conditions made the role of healthcare providers particularly challenging. We hypothesize that, in Canada, deteriorating working conditions and institutional pressures may have contributed to vaccine hesitancy and affected vaccine recommendation practices among healthcare providers.

In this context, the present study aimed to assess nurses’ vaccine confidence, attitudes and behaviours, while also examining the potential influence of their working conditions on these outcomes.

## 2 Methods

### 2.1 Study population and recruitment

Two voluntary recruitment methods were used. First, we sent an invitation email to 10,000 members of the nurses’ association of the province *Ordre des infirmières et infirmiers du Québec (OIIQ).* Only nurses who accept receiving study solicitations appear on this list. One follow-up invitation (reminder) was made according to the ethics committee guidelines. We also used Facebook adds to a sample of 44,688 nurses fitting these filters: living in Canada, aged ≥18, understanding French or English; having interests in the *Canadian nurse association or nursing*; and who studied or had a job title as a *Certified Nurse Midwife, Flight Nurse, Nurse Anesthetist Psychiatric and Mental Health Nurse, Operating Room Nurse, Nursing Assistant, Nursing Faculty, Clinic Nurse, Critical Care Nurse, Family Practice Nurse Practitioner Oncology, RN (Oncology Registered Nurse), Nursing Technician, Nursing Home Administrator, Certified Nurse Midwife (CNM), Scrub Nurse or Assistant Professor of Nursing*. All keywords used were pre-established by Facebook and we chose the ones that would be most relevant for our study sample. Inclusion criteria were nurses aged ≥18 practising in the province of Quebec who could answer either the validated French or English version of the questionnaire and included vaccination activities as part of their roles. The survey was administered online with the platform Voxco, available in English or French.

### 2.2 Questionnaire

This study is based on the original short form of the Professionals’ Vaccine Confidence and Behaviors Questionnaire (Pro-VC-Be), a validated questionnaire used to measure psychosocial factors influencing vaccine confidence and behaviour among healthcare providers(HCPs), which has also been validated in English as well as in French(Garrison et al., 2022). The short version of the Professionals’ Vaccine Confidence and Behaviors Questionnaire (Pro-VC-Be) questionnaire assesses ten key dimensions related to vaccination attitudes and behaviours among healthcare providers grouped in three first order dimensions (Table 1). The Professionals’ Vaccine Confidence and Behaviors Questionnaire (Pro-VC-Be) framework comprises ten first-order dimensions and two second-order dimensions.

**Table 1:** The 10 key dimensions of the short version of the Professionals’ Vaccine Confidence and Behaviors Questionnaire (Pro-VC-Be) **scale**(Garrison et al., 2022).

First order dimensions	Second order dimensions	Definition
Vaccine confidence	Perceived risks of vaccines	Assesses healthcare providers’ perceptions of risks associated with Vaccines
	Complacency	Measures healthcare providers’ perceptions of the usefulness of Vaccines
	Perceived benefit/risk balance	Captures how healthcare providers evaluate the balance between benefits and risks in adopting preventive behaviours.
	Perceived collective responsibility	Highlights the importance of collective commitment to vaccination.
Proactive efficacy	Commitment to vaccination	Assesses willingness to adhere to vaccination-supportive practices.
	Self-efficacy	Refers to healthcare providers’ belief in their ability (knowledge and skills) to effectively address vaccination with patients.
	Openness to patients	Measures attitudes toward vaccine-hesitant patients.
	Reluctant trust	Measure the extent to which healthcare providers might trust the vaccination system despite having concerns regarding some vaccines or the system itself
	Perceived constraints	Evaluates perceived external barriers to vaccine access.
Trust in authorities	Trust in authorities	Evaluates confidence in the information, recommendations, and actions provided by authorities (e.g., pharmaceutical companies, experts, public health, government).

The first second-order dimension, *Vaccine Confidence*, is composed of four first-order dimensions: *perceived risks associated with vaccines, complacency, perceived risk–benefit balance*, and *perceived collective responsibility*. The second-order dimension, *Proactive Efficacy*, encompasses two first-order dimensions: *commitment to vaccination* and *self-efficacy*. The remaining four first-order dimensions are *trust in authorities, openness to patients, reluctant trust*, and *perceived constraints*.

In the long-form version of the Professionals’ Vaccine Confidence and Behaviors Questionnaire (Pro-VC-Be) questionnaire, each first-order dimension was measured by multiple items. Consequently, each first-order dimension is modelled as a latent construct derived from its corresponding items, whereas each second-order dimension is specified as a higher-order latent construct composed of its respective first-order latent dimensions.

In contrast, the short-form version of the Professionals’ Vaccine Confidence and Behaviors Questionnaire (Pro-VC-Be) includes only one item per first-order dimension. Therefore, modelling first-order latent variables was not appropriate in the context of the present study. Instead, only the two second-order latent constructs were specified. These higher-order constructs were estimated using the observed indicators representing their corresponding first-order dimensions.

Apart from sociodemographic information (gender, having children under 18 years old, year of graduation, province and qualifications), the questionnaire was built in three sections: 1) *Vaccination-related behaviours*: vaccine activities (checking if patients are up-to-date with immunization), frequency of recommendation for influenza and COVID-19, frequency of recommendation for other routine vaccines, and self-vaccination against COVID-19 and influenza; 2) Professionals’ Vaccine Confidence and Behaviors Questionnaire (Pro-VC-Be) *dimensions*: immunization resources (trust in authorities, vaccine confidence and proactive efficacy), reluctant trust, perceived constraints, openness to patients. The last section was linked to the second aim of the study to assess to assess; *perceptions of working conditions* and had 7 items: work-life balance, perceived acknowledgment, working patterns, time spent with patients, remuneration, evolution of work conditions since COVID-19 and motivation to retain vaccinating roles (the questionnaire is available in Appendix 1). This section was informed by *the psychosocial risk factors in the context of work* by Karasek and Siegrist (Karasek and Theorell, 1990; Siegrist et al., 2004) as used by Le Breton and al. in a large survey in France with 18,888 nurses (Le Breton et al., 2025). This section was included based on the hypothesis that work conditions could impact immunization resources and reluctant trust among healthcare providers and also affect vaccine confidence. This adds a contextual level to the questionnaire, which helps assess systemic (social, political and cultural situations having an impact on the healthcare workforce).

## 3 Data analysis

### 3.1 Measures

For vaccination-related behaviours, the frequency of recommendation was based on a frequency 4-point Likert scale ranging from 1 (“never”) to 4 (“always”), with the option “do not know”, do not want to answer and “does not apply to my practice”. These latter options were treated as missing values. Professionals’ Vaccine Confidence and Behaviors Questionnaire (Pro-VC-Be) items and work condition items were based on a 5-point Likert scale ranging from 1 (“strongly disagree”) to 5 (“strongly agree”) with a middle “uncertain” choice. Responses of “I don’t know” were recoded 3 (“Uncertain”) and “I don’t want to answer” were treated as missing values.

### 3.2 Descriptive analyses

Descriptive statistics were generated for sociodemographic items, variables and outcomes. The vaccination-related behaviours are the four dependent variables analyzed in this study: 1) vaccine activities (checking if patients are up to date with vaccine recommendations), 2) frequency of recommendations for routine vaccines (MMR and HEP B for teenagers, DTC for pregnancy, MENC for 12 months old, HPV for 11–14 year-old boys and girls, seasonal flu for at risk populations), 3) frequency of recommendations for COVID-19 and influenza vaccines (for adults, children from 5 to 11 years old, teenagers from 12 to 17 and pregnant people), 4) vaccination status against COVID-19 and influenza all treated as continuous variables on a 0–100 mean scale. Perceived working conditions (satisfaction in work like balance, feeling that your competence is appreciated and seen, work schedule, time spent with patients, and salary) and perceived deterioration of working conditions were also included as explanatory variables (perception of degradation since the COVID-19 pandemic, thinking about quitting in the next 12 months). The explanatory variables included were vaccine confidence, trust in authorities and proactive efficacy.

### 3.3 Missing data imputation

Missing data at the item level was handled using multiple imputations for descriptive statistics and linear regressions. Predictive mean matching was applied to ordinal variables, and discriminant analysis methods were used for categorical variables. Ten imputed datasets were generated to account for uncertainty associated with the imputation process, and estimates were pooled according to Rubin’s rules. Items were imputed prior to scale construction in order to compute composite scores.

### 3.4 Regressions

Scores for each participant were constructed by summing their answers to the corresponding dimensions and dividing the sum by the number of items. Vaccine behaviour scores, based on frequency Likert scale items (from 1=never to 4=always), were linearly transformed to obtain scores ranging from 0 to 100 (items mean on a scale from 0–100: scores from 1–5: x*25–25 for score from 1–4: x*100/3–100/3), then analysed with a 75% threshold to represent nurses with very frequent vaccine recommendations and immunization activities. Higher scores indicate more positive views or attitudes toward vaccination.

Multivariate analyses were conducted following the validated approach used in previous studies using the Professionals’ Vaccine Confidence and Behaviors Questionnaire (Pro-VC-Be) short form questionnaire.(Gagneur et al., 2025; Garrison et al., 2022). Four linear regression analyses were conducted to examine the associations between each of the vaccine-related behaviours (vaccine activities, COVID-19/influenza vaccine recommendations, other routine vaccine recommendations, self-vaccinations) and all dimensions of the Professionals’ Vaccine Confidence and Behaviors Questionnaire (Pro-VC-Be) (vaccine confidence, trust in authorities, proactive efficacy and reluctant trust) which were tested separately and grouped as independent variables.

A linear regression model was used to examine the association between Professionals’ Vaccine Confidence and Behaviors Questionnaire (Pro-VC-Be) dimensions and perceived work conditions: work-life balance, perceived acknowledgment, working patterns, time spent with patients, remuneration, the evolution of work conditions since COVID-19 and motivation to retain a vaccinating role. One model assesses the association with vaccine confidence, trust in authorities, proactive efficacy and reluctant trust, while another uses the immunization s dimension.

### 3.5 Structural equation modelling

To better understand the direct and indirect relationship between dimensions measuring vaccine hesitancy, structural equation modelling (SEM) was performed following the approach proposed by Le Breton et al.(Le Breton et al., 2025), using the raw (non-imputed) dataset and full information maximum likelihood (FIML) estimation to account for missing data during model estimation.

The structural equation modeling (SEM) approach was used to evaluate the hypothesized relationships between job satisfaction, trust in authorities, vaccine confidence, vaccination-related proactive efficacy, and vaccination-related behaviours, taking into account the multiple associations between these dimensions. Model fit was assessed using the standardized root mean square residual (SRMR) and the root mean square error of approximation (RMSEA), as well as the goodness-of-fit index (GFI), adjusted goodness-of-fit index (AGFI), and Bentler comparative fit index (CFI). Model fit was considered satisfactory when standardized root mean square residual and root mean square error of approximation values were below 0.08 and goodness-of-fit index, adjusted goodness-of-fit-index and Bentler comparative fit index values exceeded 0.90. All observed indicators were significantly and positively associated with their respective first order constructs, supporting the validity of the measurement model (p < 0.05).

All analyses were conducted using SAS version 9.4.

## 4 Results

A total of n = 1428 nurses answered the survey. Of these, 108 participants were excluded because they lived outside of the province of Quebec. This was decided due to the small sample of nurses outside of Quebec in order to ensure that work organizations were similar. A total of n = 687 participants were members of the list of the Ordre des infirmières et infirmiers du Québec and the rest answered from our Facebook add (n = 633). The sociodemographic profile of our participants is presented in Table 2.

**Table 2:** Sociodemographic.

n = 1320	%
Survey answered in French	97.2
Women	90.9
Children under 18 years old living in the same home	44.1
**Years post graduation**	
<5	12.6
5 to 10	15.6
11 to 15	13.8
15+	58.0
**Qualification**	
Registered Nurse (college degree)	41.3
Clinical Nurse bachelor’s degree in nursing (BScN/BN)	54.5
Clinical Nurse Specialist (master’s degree)	2.2
Specialized Nurse Practitioner (master’s degree and specific specialization)	2.1

Table 3 presents the mean score for vaccination-related behaviours, Professionals’ Vaccine Confidence and Behaviors Questionnaire (Pro-VC-Be) dimensions and perceived work conditions. For vaccine activities (i.e., ensuring patients’ vaccines are up to date), the mean score is 53.8. Mean scores for vaccine recommendations and for influenza and COVID-19 recommendations were at 40.9 and 41.1, respectively, while the score for self-vaccination behaviours was at 64.7. The mean scores for vaccine confidence and trust in authorities are 81.5 and 80.7 respectively, and the mean score for proactive efficacy is 48.9, resulting in an overall mean immunization resources score of 63.9. For perceptions of work conditions, the mean was 56 (for a positive perception) and for perceived degradation of working conditions since the COVID-19 pandemic, the mean was 51.3 (Table 3).

**Table 3:** Mean score of the Professionals’ Vaccine Confidence and Behaviors Questionnaire (Pro-VC-Be) dimensions, vaccination behaviours and perceived working conditions.

Dimensions **	Mean (Scores /100)	95% Confidence Limits*		MIN	MAX
**Vaccine confidence** (perceived risks of vaccines, complacency, perceived benefits/risks balance, perceived collective responsibility)	81.5	80.6	82.4	0	100
**Trust in authorities**	80.7	79.5	81.9	0	100
**Proactive efficacy** (commitment to vaccination, self-efficacy)	48.9	48.1	49.6	0	100
**Reluctant trust**	50.6	48.9	52.3	0	100
**Immunization ressources** (Vaccine confidence, trust in authorities, proactive efficacy)	63.9	63.3	64.5	19.2	95.8
**Vaccine activities** (checking if patients are up to date with vaccine recommendations)	53.8	52.2	55.4	0	100
**Frequency of vaccine recommendations** (MMR and HEP B for teenagers, DTC for pregnancy, MENC for 12 months old, HPV for 11–14 year-old boys and girls, seasonal flu for at risk populations)	40.9	38.9	42.9	0	100
**Frequency of COVID-19 and influenza recommendations** (for adults, children from 5 to 11 years old, teenagers from 12–17 and pregnant people)	41.1	39.2	43.0	0	100
**Self-vaccination behaviours** (having received the flu vaccine and intention for flu and COVID next season)	64.7	62.9	66.4	0	100
**Perception of working conditions** (satisfaction in work like balance, feeling that your competence is appreciated and seen, work schedule, time spent with patients, salary)	56.0	54.9	57.1	0	100
**Perceived degradation of working conditions** (perception of degradation since the COVID-19 pandemic, thinking about quitting in the next 12 months)	51.3	50.1	52.6	0	100

* Confidence intervals for samples means accounting for data imputation, not confidence intervals for population means.

** Missing values by dimensions: vaccine confidence=147, trust in authorities=28, proactive efficacy=231, vaccine attitudes=348, vaccine activities=300, specific vaccination activities=745, COVID-19 recommendation=612, self-vaccination=180, work conditions perception=189, work degradation=94, reluctant trust=188.

Across all scales items, 2.43% of responses were “I don’t know” and 3.10% were “I prefer not to answer,” on average. The “does not apply” response accounted for a mean of 29.12% of responses across items in the first section of the scales, which addressed work-related vaccination activities. Although eligibility required participants to be involved in vaccination activities as part of their work, not all activities were applicable to every participant. The latter response option was only included in the first part of the scale, which assessed vaccine-related activities (See supplementary material for missing data frequency by questions). For the Pro-VC-Be scale, “I don’t know” responses were recoded as “3: Uncertain.” All other response options that did not correspond to a valid response were coded as missing data and handled using multiple imputation. We also included “does not apply” responses in the multiple imputation procedure, as many respondents who selected this option for certain items reported engaging in vaccination-related activities and did not consistently select “does not apply” across all items.

### 4.1 Regressions

The models account for the uncertainty associated with multiple imputation. All predictors included in each model were entered simultaneously rather than separately. Regression coefficients are unstandardized (i.e., raw coefficients), and the models were not adjusted for professional variables. The outcome variables were analyzed as continuous variables and were not dichotomized.

Two sets of models were examined. The first set included vaccine confidence, vaccine hesitancy, trust in authorities, and proactive efficacy as simultaneous predictors. The models explained 23.2% of the variance in vaccine activity frequency (R² = 0.232), 16.4% in the frequency of specific recommendations (R² = 0.164), 25.0% in the frequency of COVID-19 recommendations (R² = 0.250), 24.5% in influenza/COVID-19 self-vaccination (R² = 0.245), 5.6% in perceived working conditions (R² = 0.056), and 3.7% in perceived degradation of working conditions (R² = 0.037).

The second set of models included total vaccine attitudes as the sole predictor. These models explained 14.4% of the variance in vaccine activity frequency (R² = 0.144), 10.2% in the frequency of specific recommendations (R² = 0.102), 23.3% in the frequency of COVID-19 recommendations (R² = 0.233), 25.8% in influenza/COVID-19 self-vaccination (R² = 0.258), 4.3% in perceived working conditions (R² = 0.043), and 3.3% in perceived degradation of working conditions (R² = 0.033). These clarifications have been added to the revised manuscript.

### 4.2 Determinants of nurses’ vaccination behaviours: professionals’ vaccine confidence and behaviors questionnaire (Pro-VC-Be)

There was a statistically significant association between the score of immunization and vaccine activities, which indicated that higher immunization resources were associated with higher vaccine activities (β = 0.96, 95% confidence interval: [0.8349;1.086752]). Immunization resources were also associated with vaccine recommendations (β =0.93[0.7712;1.08922]), the frequency of COVID-19 vaccine recommendations (β =1.33[1.1835;1.4816]) and self-vaccination behaviours for COVID-19 and influenza (β =1.38[1.2514;1.5004]). All regression coefficients were highly significant (p<.0001). Thus, the more positive the attitude towards vaccination, the higher the positive vaccination behaviour.

Three out of the four dimensions of the Professionals’ Vaccine Confidence and Behaviors Questionnaire (Pro-VC-Be) were significantly associated with higher vaccination-related activities (vaccine confidence: β=0. 0.24 [0.1300; 0.35767]; proactive efficacy: β=0.85[0.7421; 0.96230]) and reluctant trust (β=0.057[0.0052; 0.10939]. Vaccine confidence and proactive efficacy were positively associated with higher scores of vaccine recommendations (vaccine confidence: β=0.35[0.1964 ;0.5022]; proactive efficacy: β=0.85[0.6998;0.9962]). Furthermore, vaccine confidence (β=0.38[0.2508; 0.5053]), proactive efficacy (β=0.79[0.667;0.9037]) and trust in authorities (β=0.17[0.0639; 0.2671] were positively associated with COVID-19 and influenza recommendations. Finally, vaccine confidence (β=0.57[0.4509;0.69069]) and trust in authorities (β=0.32[0.2371;0.41152]) were positively associated with self-vaccination behaviours.

No significant association was found between trust in authorities and vaccine activities’ scores, reluctant trust or trust in authorities were not associated with vaccine recommendations, reluctant trust was not associated with COVID-19 and influenza recommendations, and reluctant trust nor proactive efficacy were not associated with self-vaccination behaviour.

### 4.3 Nurses work conditions association with professionals’ vaccine confidence and behaviors questionnaire (Pro-VC-Be) scores

Perceived working conditions and perceived degradation in working conditions since the COVID-19 pandemic were also analyzed. Significant positive associations were observed between trust in authorities (β = 0.16 [0.09572, 0.22212]) and proactive efficacy (β = 0.18 [0.09909, 0.26508]) and perceived working conditions. Lower levels of trust in authorities and lower proactive efficacy were associated with more negative perceptions of working conditions. In addition, trust in authorities (β=−0.14 [−0.21174; −0.06245], proactive efficacy: (β=−0.12 [−0.21840; −0.02299]) and reluctant trust (β= 0.05[0.00003; 0.09291] were significantly associated with deterioration in working conditions since the COVID-19 pandemic.

No significant association was found between vaccine confidence and reluctant trust with perception of working conditions, and neither between vaccine confidence with perception of work degradation since the COVID-19 pandemic.

There was a significant association (p<.0001) between immunization resources and both perceptions of working conditions (β=0.36[0.27010; 0.45199]) and the deterioration of working conditions since the COVID-19 pandemic (β=−0.37 [−0.47223; −0.25847]). Thus, the more positive the attitude towards vaccination, the more positive the perception of working conditions, and the fewer working conditions were perceived to have deteriorated because of the COVID-19 pandemic.

## 5 Structural equation model

The model showed good fit to the data, with a Standardized Root Mean Square Residual of 0.0409, a Goodness-of-Fit Index of 0.9637, an Adjusted Goodness-of-Fit Index of 0.9497, a Root Mean Square Error of Approximation of 0.0447, and a Comparative Fit Index of 0.9571. The Standardized Root Mean Square Residual and Root Mean Square Error of Approximation were below 0.08, while the Goodness-of-Fit Index, Adjusted Goodness-of-Fit Index, and Comparative Fit Index were above 0.90, indicating an acceptable-to-good model fit.

Full-information maximum likelihood estimation was used to account for missing data in the model estimation. The model examined the effects of satisfaction with working conditions on trust in authorities and vaccine recommendations; the effects of trust in authorities on proactive efficacy and vaccine confidence; the effect of vaccine confidence on vaccine recommendations; and the effect of proactive efficacy on vaccine recommendations.

All constructs included in the structural model were specified as latent variables, with their corresponding questionnaire items serving as indicators. A unit variance was fixed for each latent construct for model identification. Indirect effects between latent variables were calculated as the sum of the products of the path coefficients along all relevant indirect pathways. For example, the indirect effect of satisfaction with working conditions on vaccine confidence was calculated as 0.22826 × 0.99174 = 0.2264. Similarly, the indirect effects of satisfaction with working conditions on proactive efficacy and vaccine recommendations were 0.1330 and 0.1080, respectively, while the indirect effect of trust in authorities on vaccine recommendations was 0.4733.

Direct association (represented as single-headed arrows in Fig. 1) define direct hypotheses, showing how one variable immediately impacts another. An indirect effect (or indirect associations, represented with dotted arrows) occurs when one variable is associated with another through other variables, called a mediators. Proactive efficacy was associated with vaccine recommendations with a direct association estimate following the structural equation modeling (SEM) analysis of 0.7 (p < 0.0001) (Fig. 1). Associations were observed between working conditions and trust in authorities (β=0.23 p < 0.0001). Strong standardized regression coefficients were observed between trust in authorities and vaccine confidence (0.99 p < 0.0001).

**Fig. 1:**
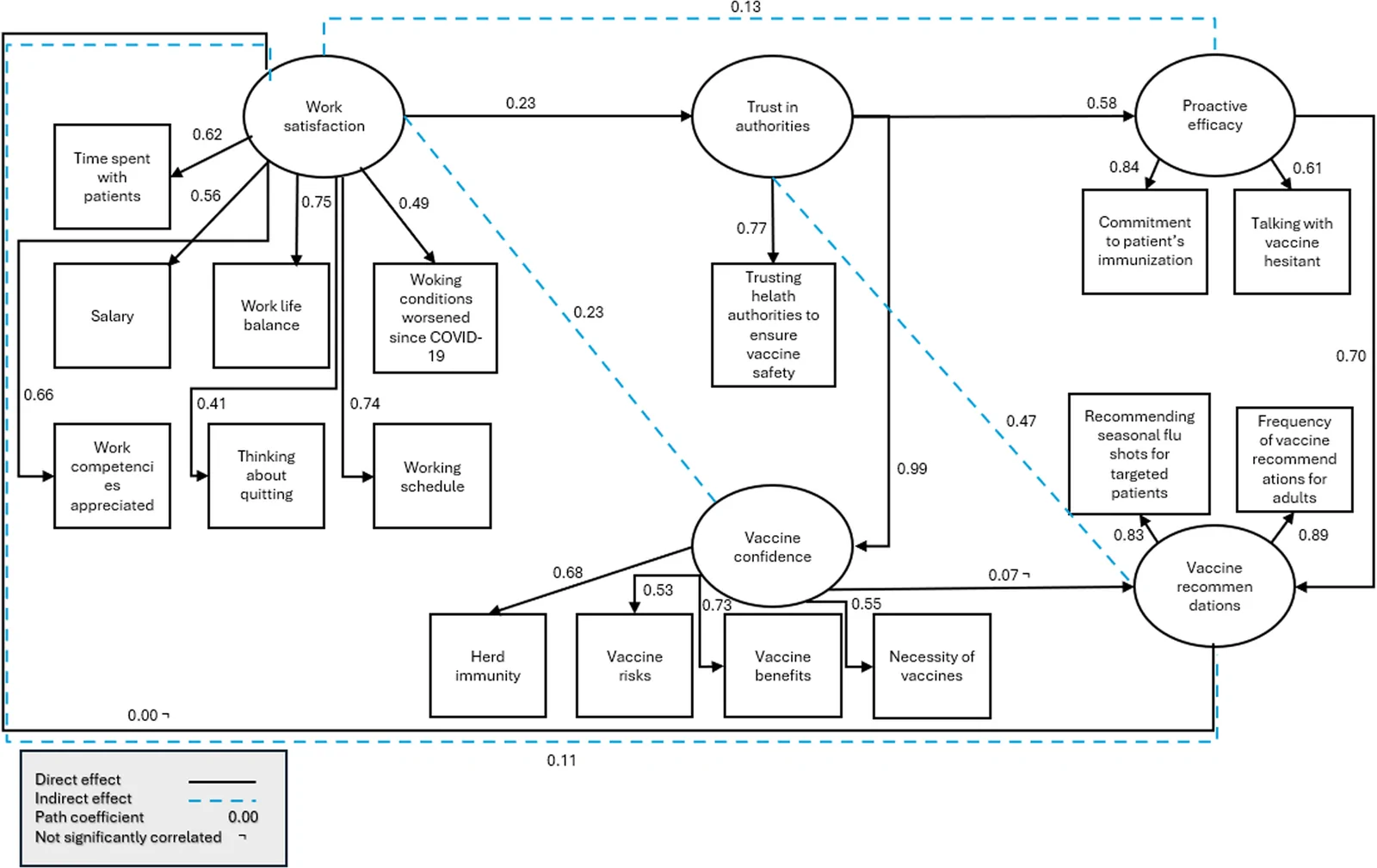
Association Model Between Work Satisfaction and Professionals’ Vaccine Confidence and Behaviors Questionnaire (Pro-VC-Be) dimensions.

No significant direct relationship between vaccine confidence and vaccine recommendations were observed. There was no significant direct association between job satisfaction and vaccine recommendations.

Furthermore, strong indirect correlations (p < 0.0001) were found between work satisfaction and confidence in vaccines (β =0.23), satisfaction with working conditions and proactive efficacy (β =0.13), satisfaction with working conditions and vaccine recommendations (β =0.11), and between trust in authorities and vaccine recommendations (β =0.47). All regression parameters were positive.

## 6 Discussion

These results contribute to a growing body of evidence demonstrating that vaccine hesitancy is present among nurses, despite their critical role in promoting public vaccination and protecting populations. Moreover, findings of this study conducted with Quebec nurses indicate that vaccine confidence and trust in authorities were high. Vaccine hesitancy in healthcare providers has been documented across multiple countries and professional groups, with varying prevalence and determinants depending on context and vaccine type(Gagneux-Brunon et al., 2024; Le Breton et al., 2025; Peterson et al., 2022b).

The very strong standardized association observed between trust in authorities and vaccine confidence suggests that these two constructs are closely interconnected among nurses. This finding is consistent with previous research identifying trust in health authorities and institutions as an important component of vaccine confidence and vaccine decision-making(Brownlie and Howson, 2006). However, the magnitude of the association should be interpreted with caution. Because trust in authorities and vaccine confidence were assessed using self-reported items within the same questionnaire, shared measurement characteristics and common method variance may have contributed to the strength of the observed relationship. Moreover, the near-unit coefficient may indicate substantial conceptual overlap between the two latent constructs, which should be considered when interpreting their distinct contributions to vaccine-related outcomes. Among healthcare workers, lower trust in health authorities, public health policies, and the broader healthcare system have been consistently associated with greater vaccine hesitancy(Ahmad et al., 2022). Lower trust in health authorities may undermine healthcare workers' confidence in the legitimacy, transparency, and reliability of vaccine-related recommendations. This erosion of institutional trust can increase uncertainty regarding vaccine safety and effectiveness, reduce adherence to public health guidance, and foster greater receptivity to alternative or conflicting sources of information, ultimately contributing to vaccine hesitancy(Peterson et al., 2022c). Empirical studies show that confidence in institutions and public health measures is a key predictor of vaccine acceptance, while distrust contributes to refusal and skepticism, even within medically trained populations (Huang et al., 2025; Khubchandani et al., 2022). These associations between trust in health authorities, vaccine confidence and recommendations were also shown in our study. Perceptions of working conditions and their perceived degradation since the COVID-19 pandemic were also influential in nurses’ behaviours in vaccine recommendations.

Professional roles and occupations also shape vaccine hesitancy(Browne et al., 2022). Several studies show differences in hesitancy by occupation within health care settings: nurses and non-physician personnel often exhibit higher hesitancy compared to physicians, potentially reflecting differences in educational background, exposure to vaccination science, or workplace culture(Kaur et al., 2023; Rosser et al., 2024).

The implications of vaccine hesitancy among nurses extend beyond individual protection against diseases to broader public health outcomes. As trusted sources of health information, healthcare providers vaccine confidence influences patient attitudes and community uptake(Lin et al., 2021b; Paterson et al., 2016b). Vaccine hesitancy among healthcare providers may undermine public confidence in immunization programs, reduce uptake of recommended vaccines, and weaken efforts to achieve collective immunity(ASSET, 2017).

Our results also point to the importance of considering the work environment and professional experiences of healthcare providers. Political, structural, and sanitary contexts such as a pandemic shape vaccine confidence and trust among the general population, and nurses are not exempt from these influences(Ma et al., 2025). However, our findings are coherent with the 2025 study from Le Breton using the short form questionnaire with the section on work condition in France and suggest that the occupational and institutional context of nursing may amplify these dynamics(Le Breton et al., 2025). The proposed effects of improving working conditions are potentially important, but they remain to be tested in longitudinal or intervention studies. Beyond the direct clinical burden of the pandemic reported in the literature, nurses reported experiences of chronic understaffing, inadequate personal protective equipment, forced overtime, and limited institutional support and is coherent with the Canadian and French contextual experiences (Gamble et al., 2022a). These experiences may have eroded nurses' trust not only in their employing institutions, but also in broader public health authorities whose recommendations and mandates were experienced as disconnected from the realities of front-line care(Gamble et al., 2022b). In this context, the disconnect between personal confidence in vaccines and active recommendation behaviour observed in our sample may therefore reflect not a lack of belief in immunization, but rather a form of professional disengagement rooted in structural dissatisfaction(Delgado-Ron et al., 2024; Gagneur et al., 2025). The confidence–recommendation gap may reflect professional disengagement associated with structural dissatisfaction, although this interpretation remains tentative and the gap may also arise from differences in professional roles, vaccination-related responsibilities, and the applicability of specific items to respondents’ practice contexts.

Conversely, our results also suggest a positive pathway: nurses who perceived their working conditions more favourably reported greater trust in health authorities and indirectly exhibited stronger vaccine recommendation behaviour. This finding is consistent with broader literature on organizational trust, which demonstrates that employees who feel valued, supported, and recognized by their institutions are more likely to align their behaviour with institutional goals and recommendations(Kurtessis et al., 2017). Applied to the vaccination context, this implies that improvements in working conditions, meaningful recognition of nurses' contributions, reduced administrative burden, and genuine consultation in policy development may translate into measurable gains in vaccine promotion behaviours at the point of care(Ricciardelli and MacDonald, 2022).

## 7 Limitations

This study has several limitations inherent to its cross-sectional design. First, as exposures and outcomes were measured at a single point in time, causal relationships cannot be inferred. In addition, the use of self-reported data may have introduced information bias, including recall bias and social desirability bias. The heterogeneity of the sample due to voluntary sample from two recruitment methods also confers limitations in regard to the generalisation of the results and may also induce a selection bias. Furthermore, cross-sectional designs do not allow for the assessment of temporal changes in attitudes, behaviours, or exposures, thereby limiting the ability to examine trajectories or long-term effects. Selection bias may also have occurred, as participants were primarily recruited through a voluntary list of members of the Ordre des infirmières et infirmiers du Québec. Although efforts were made to obtain a sociodemographically diverse sample, the recruitment strategy may limit representativeness. Finally, the relatively small sample size restricts the generalizability of the findings to the broader population of nurses in the province. It may also have affected the precision of the estimates, and some associations could be over- or underestimated. Therefore, the results should be interpreted with caution.

## 8 Conclusions

As Canada just lost its measles elimination status, it is clear that goals in terms of vaccine coverage rates targeted by health care authorities are not met in Quebec(Taylor, 2025). Given the critical role of healthcare providers in promoting vaccination, addressing the structural and occupational factors that shape their recommendation behaviours is essential. Our findings should also be considered in the broader context of the structural and workplace conditions in which nurses provide care. Vaccine attitudes and behaviors may have been influenced not only by individual levels of confidence or trust, but also by the prolonged psychological and occupational impacts of the COVID-19 pandemic, persistent workforce shortages, increased workload, and challenging working conditions. In some settings, nurses may also face increasing physical and psychological risks in the workplace, which could shape their perceptions of vaccines, health authorities, and their professional role. Although these factors were not directly assessed in the present study, they represent important contextual influences that should be considered when interpreting nurses’ vaccine attitudes and behaviors. Interventions aimed at improving vaccine uptake have traditionally focused on patient-facing communication and education. Our findings suggest that equal attention should be paid to the institutional and occupational conditions under which healthcare providers operate. Investing in the well-being and professional recognition of nurses may be a meaningful lever for strengthening immunization programs at the population level. Future research should examine the causal mechanisms underlying the relationship between working conditions, institutional trust, and vaccine recommendation behaviour, including the potential mediating roles of moral injury and also examine how organizational and working conditions interact with individual-level determinants of vaccine confidence and hesitancy.

## Declaration of generative AI and AI-assisted technologies in the manuscript preparation process

Translation tools from French to English such as Deepl have been partly used to ensure proper English phrasing when needed. Rephrasing Antidote tools also have been lightly used. Copilot helped partly with literature review as a search engine. After using those tools, the authors reviewed and edited the content as needed and takes full responsibility for the content of the published article.

## Registration

Not registered

## Ethical approval and informed consent

This study has been approved by the ethics committee of Centre Hospitalier Universitaire de Québec (MP-20–2023–6447). Data collection occurred between 2023–03–31 and 2024–01–31 No nominal information was collected in the survey. Participation was voluntary and measures taken to preserve confidentiality and data protection were taken. Nurses received a solicitation email with information about the survey, including confidentiality and data protection and were able to pursue answering the questionnaire.

## Ethics declaration

Written informed consent to take part in the study and to publish the article has been obtained from all participants or their legal representatives. The privacy rights of participants have been observed.

This study was performed in compliance with relevant laws, regulatory frameworks and guidelines where the research took place. This study was approved by the CER du CHU de Québec-Université Laval. (Approval No 2023–6815)

## Funding

This project was conducted by the CIHR Applied Public Health Chair on the Anthropology of Vaccination with funding from the Canadian Institute of Health Research.

## CRediT authorship contribution statement

**Eve Dubé:** Writing – review & editing, Investigation, Funding acquisition, Conceptualization. **Marie-Eve Trottier:** Writing – review & editing, Writing – original draft, Investigation, Formal analysis. **Lisa Fressard:** Writing – review & editing, Formal analysis. **Philip White:** Writing – review & editing. **Arnaud Gagneur:** Writing – review & editing, Conceptualization. **Pierre Verger:** Writing – review & editing, Methodology, Conceptualization.

## Declaration of competing interest

The authors have no competing interest to disclose.
