# Supplementary material for: Assessing vaccine hesitancy among nurses in Quebec, Canada using the professionals’ vaccine confidence and behaviors questionnaire: a cross sectional survey
Source: Int J Nurs Stud Adv. 2026 Oct 27;11:100722. doi: 10.1016/j.ijnsa.2026.100722 (PMC13755451; doi:10.1016/j.ijnsa.2026.100722)
Supplement: Supplementary file 1 [file mmc1.docx]

**Supplementary material: Missing value repartition across the PRO-VC-Be scale**

For the patients you treat and for whom vaccination is recommended according to official guidelines:

| **How often do you check that your patients have received the vaccines that are recommended for them?** | **Frequency** | **Percent** |
| --- | --- | --- |
| I don’t know | 7 | 0.49 |
| I prefer not to answer | 5 | 0.35 |
| Not applicable | 171 | 12.00 |

| **How often do you check if your patients are up to date with vaccination?** | **Frequency** | **Percent** |
| --- | --- | --- |
| I don’t know | 6 | 0.42 |
| I prefer not to answer | 5 | 0.35 |
| Not applicable | 210 | 14.74 |

| **How often do you recommend the vaccines that are indicated for them?** | **Frequency** | **Percent** |
| --- | --- | --- |
| I don’t know | 7 | 0.49 |
| I prefer not to answer | 11 | 0.77 |
| Not applicable | 185 | 12.98 |

**How often do you recommend the following vaccines:**

| **Measles mumps and rubella (MMR) for unvaccinated teenagers?** | **Frequency** | **Percent** |
| --- | --- | --- |
| I don’t know | 29 | 2.04 |
| I prefer not to answer | 8 | 0.56 |
| Not applicable | 572 | 40.14 |

| **Whooping cough during pregnancy?** | **Frequency** | **Percent** |
| --- | --- | --- |
| I don’t know | 25 | 1.75 |
| I prefer not to answer | 3 | 0.21 |
| Not applicable | 582 | 40.84 |

| **Meningitis C for 12 months old?** | **Frequency** | **Percent** |
| --- | --- | --- |
| I don’t know | 19 | 1.33 |
| I prefer not to answer | 7 | 0.49 |
| Not applicable | 613 | 43.02 |

| **Human papilloma virus (HPV) for 11- to 14-year-old boys and girls?** | **Frequency** | **Percent** |
| --- | --- | --- |
| I don’t know | 11 | 0.77 |
| I prefer not to answer | 7 | 0.49 |
| Not applicable | 586 | 41.12 |

| **Hepatitis B for unvaccinated teenagers?** | **Frequency** | **Percent** |
| --- | --- | --- |
| I don’t know | 14 | 0.98 |
| I prefer not to answer | 12 | 0.84 |
| Not applicable | 586 | 41.12 |

| **Seasonal flu for adults younger than 65 years old living with a chronic disease?** | **Frequency** | **Percent** |
| --- | --- | --- |
| I don’t know | 3 | 0.21 |
| I prefer not to answer | 7 | 0.49 |
| Not applicable | 225 | 15.79 |

How often do you recommend COVID-19 vaccines for:

| **Adults?** | **Frequency** | **Percent** |
| --- | --- | --- |
| I don’t know | 11 | 0.77 |
| I prefer not to answer | 16 | 1.12 |
| Not applicable | 178 | 12.49 |

| **Children from 5 to 11 years old?** | **Frequency** | **Percent** |
| --- | --- | --- |
| I don’t know | 15 | 1.05 |
| I prefer not to answer | 16 | 1.12 |
| Not applicable | 507 | 35.58 |

| **Teenagers from 12 to 17 years old?** | **Frequency** | **Percent** |
| --- | --- | --- |
| I don’t know | 12 | 0.84 |
| I prefer not to answer | 15 | 1.05 |
| Not applicable | 507 | 35.58 |

| **Pregnant people?** | **Frequency** | **Percent** |
| --- | --- | --- |
| I don’t know | 26 | 1.82 |
| I prefer not to answer | 17 | 1.19 |
| Not applicable | 472 | 33.12 |

How much do you agree with the following statements?

| **There are no serious risks associated with vaccines in general** | **Frequency** | **Percent** |
| --- | --- | --- |
| I don’t know | 6 | 0.42 |
| I prefer not to answer | 20 | 1.40 |

| **ome vaccines that are recommended by authorities are not necessary because the diseases that they prevent are mild** | **Frequency** | **Percent** |
| --- | --- | --- |
| I don’t know | 18 | 1.26 |
| I prefer not to answer | 33 | 2.32 |

| **Vaccine benefits are much higher than vaccine risks** | **Frequency** | **Percent** |
| --- | --- | --- |
| I don’t know | 19 | 1.33 |
| I prefer not to answer | 20 | 1.40 |

| **I recommend vaccines indicated to my patients because vaccination protects the whole population (herd immunity)** | **Frequency** | **Percent** |
| --- | --- | --- |
| I don’t know | 43 | 3.02 |
| I prefer not to answer | 119 | 8.35 |

| **I trust health authorities to ensure vaccine safety** | **Frequency** | **Percent** |
| --- | --- | --- |
| I don’t know | 18 | 1.26 |
| I prefer not to answer | 28 | 1.96 |

| **The cost of some vaccines is an issue for some patients and it can make me hesitate to recommend them.** | **Frequency** | **Percent** |
| --- | --- | --- |
| I don’t know | 206 | 14.46 |
| I prefer not to answer | 128 | 8.98 |

| **I give information about vaccine risks and benefits to my patients but I let them make their own decisions without influencing them.** | **Frequency** | **Percent** |
| --- | --- | --- |
| I don’t know | 39 | 2.74 |
| I prefer not to answer | 100 | 7.02 |

| **I am committed to my patient being immunized** | **Frequency** | **Percent** |
| --- | --- | --- |
| I don’t know | 66 | 4.63 |
| I prefer not to answer | 155 | 10.88 |

| **I feel adequately trained on how to talk about vaccines to vaccine hesitant patients.** | **Frequency** | **Percent** |
| --- | --- | --- |
| I don’t know | 39 | 2.74 |
| I prefer not to answer | 80 | 5.61 |

| **I recommend indicated vaccines even if the reason behind the policy is unclear to me.** | **Frequency** | **Percent** |
| --- | --- | --- |
| I don’t know | 139 | 9.75 |
| I prefer not to answer | 188 | 13.19 |

| **Mandatory COVID-19 vaccination for health care providers is a good thing** | **Frequency** | **Percent** |
| --- | --- | --- |
| I don’t know | 20 | 1.40 |
| I prefer not to answer | 33 | 2.32 |

| **Alternative medical therapies are effective for treating diseases** | **Frequency** | **Percent** |
| --- | --- | --- |
| I don’t know | 76 | 5.33 |
| I prefer not to answer | 56 | 3.93 |

Are you satisfied about :

| **Work life balance?** | **Frequency** | **Percent** |
| --- | --- | --- |
| I don’t know | 14 | 0.98 |
| I prefer not to answer | 32 | 2.25 |

| **The appreciation of your competencies at your workplace or in your work environment?** | **Frequency** | **Percent** |
| --- | --- | --- |
| I don’t know | 22 | 1.54 |
| I prefer not to answer | 26 | 1.82 |

| **Your work schedules?** | **Frequency** | **Percent** |
| --- | --- | --- |
| I don’t know | 5 | 0.35 |
| I prefer not to answer | 36 | 2.53 |

| **Time spent with patients?** | **Frequency** | **Percent** |
| --- | --- | --- |
| I don’t know | 25 | 1.75 |
| I prefer not to answer | 84 | 5.89 |

| **Your salary?** | **Frequency** | **Percent** |
| --- | --- | --- |
| I don’t know | 14 | 0.98 |
| I prefer not to answer | 36 | 2.53 |

Do you agree with the following statements:

| **Your working conditions have worsened since the COVID-19 pandemic** | **Frequency** | **Percent** |
| --- | --- | --- |
| I don’t know | 38 | 2.67 |
| I prefer not to answer | 42 | 2.95 |

| **You think about quitting your job in the next 12 months.** | **Frequency** | **Percent** |
| --- | --- | --- |
| I don’t know | 116 | 8.14 |
| I prefer not to answer | 68 | 4.77 |

| **Have you received seasonal flu this year?** | **Frequency** | **Percent** |
| --- | --- | --- |
| I do not remember | 20 | 1.40 |
| I prefer not to answer | 27 | 1.89 |

| **Do you have the intention of being vaccinated against seasonal flu next year?** | **Frequency** | **Percent** |
| --- | --- | --- |
| I don’t know | 67 | 4.70 |
| I prefer not to answer | 16 | 1.12 |

| **Will you get immunized against COVID-19 for next season?** | **Frequency** | **Percent** |
| --- | --- | --- |
| I don’t know | 96 | 6.74 |
| I prefer not to answer | 16 | 1.12 |
